# *PIK3CA* mutations can initiate pancreatic tumorigenesis and are targetable with PI3K inhibitors

**DOI:** 10.1038/oncsis.2015.28

**Published:** 2015-10-05

**Authors:** S N Payne, M E Maher, N H Tran, D R Van De Hey, T M Foley, A E Yueh, A A Leystra, C A Pasch, J J Jeffrey, L Clipson, K A Matkowskyj, D A Deming

**Affiliations:** 1 University of Wisconsin Carbone Cancer Center, Madison, WI, USA; 2 Division of Hematology and Oncology, Department of Medicine, University of Wisconsin, Madison, WI, USA; 3 Department of Oncology, University of Wisconsin, Madison, WI, USA; 4 Department of Pathology and Laboratory Medicine, University of Wisconsin, Madison, WI, USA; 5 William S Middleton Memorial Veterans Hospital, Madison, WI, USA

## Abstract

Aberrations in the phosphoinositide 3-kinase (PI3K) signaling pathway have a key role in the pathogenesis of numerous cancers by altering cell growth, metabolism, proliferation and apoptosis. Interest in targeting the PI3K signaling cascade continues, as new agents are being clinically evaluated. *PIK3CA* mutations result in a constitutively active PI3K and are present in a subset of pancreatic cancers. Here we examine mutant *PIK3CA*-mediated pancreatic tumorigenesis and the response of *PIK3CA* mutant pancreatic cancers to dual PI3K/mammalian target of rapamycin (mTOR) inhibition. Two murine models were generated expressing a constitutively active PI3K within the pancreas. An increase in acinar-to-ductal metaplasia and pancreatic intraepithelial neoplasms (PanINs) was identified. In one model these lesions were detected as early as 10 days of age. Invasive pancreatic ductal adenocarcinoma developed in these mice as early as 20 days of age. These cancers were highly sensitive to treatment with dual PI3K/mTOR inhibition. In the second model, PanINs and invasive cancer develop with a greater latency owing to a lesser degree of PI3K pathway activation in this murine model. In addition to PI3K pathway activation, increased ERK1/2 signaling is common in human pancreatic cancers. Phosphorylation of ERK1/2 was also investigated in these models. Phosphorylation of ERK1/2 is demonstrated in the pre-neoplastic lesions and invasive cancers. This activation of ERK1/2 is diminished with dual PI3K/mTOR inhibition. In summary, *PIK3CA* mutations can initiate pancreatic tumorigenesis and these cancers are particularly sensitive to dual PI3K/mTOR inhibition. Future studies of PI3K pathway inhibitors for patients with *PIK3CA* mutant pancreatic cancers are warranted.

## Introduction

Pancreatic adenocarcinoma is one of the leading causes of cancer-related death despite advances in cytotoxic chemotherapeutic regimens, including FOLFIRINOX and gemcitabine/nab-paclitaxel.^1, 2, 3^ The 5-year survival rate for patients with metastatic pancreatic adenocarcinoma is <1%.^4^ Recent successes in targeted therapies have occurred in some cancer types when utilizing these agents in the setting of certain oncogenes.^5, 6, 7^ Genomic profiling of tumor tissues to guide therapy continues to expand and is becoming standard in many clinical trials. A large armamentarium of targeted therapies are in clinical development, but there remains a critical need to identify how to utilize the mutation profile to predict benefits from these therapies. This is especially true for pancreatic cancers. The majority of patients with metastatic pancreatic cancer are treated in a very similar fashion despite significant advances in our understanding of tumor biology. One limiting factor in the development of targeted therapies for patients with pancreatic cancers is that most cancers are initiated secondary to a mutation in the *KRAS* gene.^8, 9, 10, 11, 12^ Murine models have been vital to establishing the role of mutant *KRAS* in pancreatic metaplasia, premalignant lesions and invasive ductal adenocarcinoma.^13, 14, 15, 16^ Unfortunately, to date an effective means to target mutant KRAS has yet to be established.

An approach to identify potentially targetable subpopulations of pancreatic cancers is to examine other mutations that may be able to initiate pancreatic tumorigenesis independent of KRAS. The *PIK3CA* gene encodes the p110α catalytic subunit of phosphoinositide 3-kinase (PI3K). *PIK3CA* mutations are common in multiple human cancers.^17^ These mutations produce a constitutively active form of the PI3K protein, which results in the phosphorylation of multiple downstream targets responsible for a wide variety of vital cellular functions.^18, 19^ PI3K initiates this signaling pathway through the phosphorylation of phosphatidylinositol 4,5-bisphosphate to phosphatidylinositol 3,4,5-triphosphate. Phosphatidylinositol 3,4,5-triphosphate then activates the serine/threonine kinase AKT, which phosphorylates multiple downstream targets. One of the prominent targets is mammalian target of rapamycin (mTOR), a serine/threonine kinase that is an important regulator of cell growth and metabolism. This kinase then mediates activation of the eukaryotic translation initiation factor 4E-binding protein (4EBP1) and the p70S6 ribosomal kinase (RPS6) that are involved in protein synthesis.

*PIK3CA* mutations have been shown to be present in 3–5% of patients with pancreatic cancer.^20, 21, 22, 23^ Though this subtype does not represent the majority of pancreatic cancers, these patient's tumors might be exquisitely sensitive to treatments targeting the PI3K pathway. In the setting of a malignancy with such dismal treatment responses and survival, the discovery of a subtype of pancreatic adenocarcinoma that might be treatable is worthy of further investigation. Here we investigate the effects of a constitutively active PI3K in the pancreas and examine the response of *PIK3CA* mutant pancreatic cancer to dual PI3K/mTOR inhibition.

## Results

### Pc^1^ Pik3ca^p110*^mice become moribund at a young age secondary to pancreatic tumors

**Figure 1:**
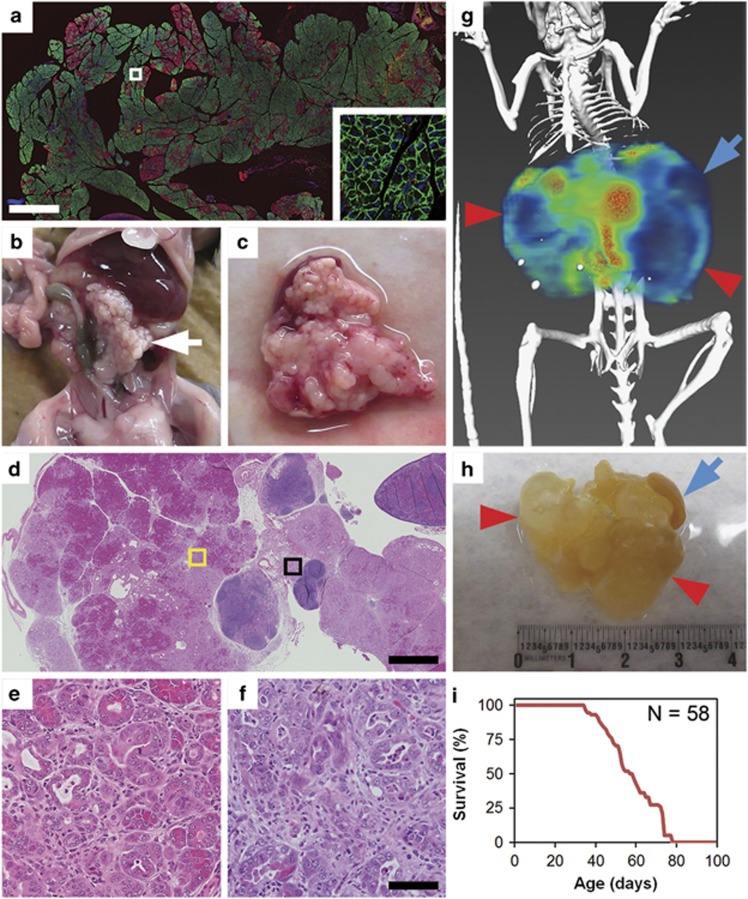
The expression of a constitutively active PI3K resulting in pancreatic tumor formation. (**a**) *Pc*^*1*^ *mT/mG*^*1*^ mice demonstrated the expression of GFP throughout the pancreas, indicating expression of the Cre recombinase at the site of interest. Size bar, 1 mm. Area outlined is shown enlarged × 20 in inset. *Pc*^*1*^ *Pik3ca*^p110*^ mice were allowed to age until moribund; at necropsy large pancreatic tumors were identified: (**b**) *in vivo*; (**c**) whole mount. (**d**) Histological analysis demonstrated heterogenous masses comprised of dysplatic acinar cells, neoplastic changes, abundant stromal infiltration and immune infiltrates. Size bar, 1mm. Area outlined in yellow is enlarged × 20 in **e**; the area outlined in black is enlarged × 20 in **f**. At higher magnification, acinar-to-ductal metaplasia could be identified (**e**) in addition to invasive pancreatic ductal adenocarcinoma (**f**). Size bar for **e** and **f**, 50 μm. (**g** and **h**) Dual hybrid ^18^F-FDG microPET/CT imaging was used to better understand the complex heterogeneity within these tumors including areas of dense fibrosis with invasive cancer (orange) and areas of cystic dilation (red arrows). Blue arrows denote the spleen. (**i**) All *Pc*^*1*^ *Pik3ca*^p110*^ mice developed tumors and became moribund at a median age of 53 days.

The effects of a constitutively active PI3K in the pancreas were investigated using genetically engineered mice. Cre was expressed in the pancreas under the control of the *PDX-1* promoter.^15^ The pattern of Cre expression was determined using a fluorescent reporter with the majority of the pancreas demonstrating green fluorescence indicating the presence of Cre (Figure 1a). *Pc*^*1*^ mice were crossed with *Pik3ca*^p110*^ mice, which carry a conditional allele encoding for a fusion protein resulting in a constitutively active PI3K.^24^ *Pc*^*1*^*Pik3ca*^p110*^ mice were found to develop large pancreatic masses (Figures 1b and c). These tumors measured 1–4 cm in size and were often associated with large cystic structures (Supplementary Figure S1). Histological sectioning confirmed large pancreatic tumors (Figure 1d) containing metaplastic acinar cells (Figure 1e) and invasive pancreatic ductal adenocarcinoma (Figure 1f). Invasive ductal adenocarcinoma was identified in over 90% of mice when moribund. ^18^F-fluorodeoxyglucose (^18^F-FDG) micro positron emission tomography/computed tomography (μPET/CT) imaging was utilized to interrogate the structural heterogeneity of these tumors. Imaging demonstrated large cystic lesions with low PET avidity and dense areas of invasive cancer with increased avidity (Figures 1g and h). All *Pc*^*1*^ *Pik3ca*^p110*^ mice became moribund owing to pancreatic cancers at a median age of 53 days (*n*=58, range 35–79 days; Figure 1i). The pancreatic parenchyma was remarkable for extensive acinar-to-ductal metaplasia, multifocal cystic dilation of the ducts, and within areas of invasive adenocarcinoma there were abortive tubular structures with nuclear atypia, an infiltrative growth pattern, increased mitotic figures and an associated desmoplastic reaction of the adjacent stroma. Biliary obstruction was likely a common cause of mortality as dense tumor tissue was often present near the common bile duct and dilation of the proximal bile duct was often encountered.

### p110* causes activation of the PI3K signaling cascade, increased cellular proliferation and dense stromal infiltration

**Figure 2:**
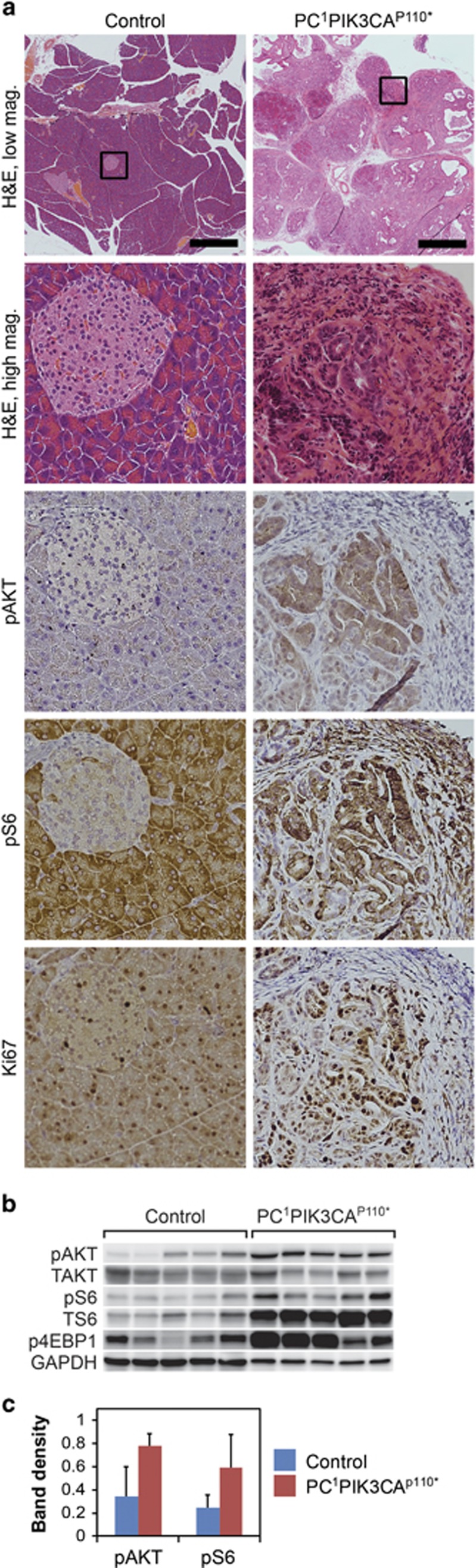
p110* results in increased activation of the PI3K pathway in the pancreas of *Pc*^*1*^ *Pik3ca*^p110*^ mice compared with age-matched controls. (**a**) A marked difference in pancreatic morphology is noted with the development of invasive pancreatic cancer surrounded by a dense fibrous infiltrate. The areas of neoplasia had increased phosphorylation of AKT and RPS6 on IHC. An increase in cellular proliferation was also observed based on an increase in nuclear Ki67 staining in the neoplastic cells. Size bar for the top panels, 1 mm. Each lower panel is a × 10 enlargement of the outlined area in the top panel in its respective column or equivalent area in the same sample. (**b**) The activation of the PI3K pathway in the pancreatic tissue of moribund *Pc*^*1*^ *Pik3ca*^p110*^ mice was also confirmed with immunoblotting. (**c**) Statistically significant increases in phosphorylation of AKT and RPS6 were identified (*P*=0.02 and *P*=0.03, respectively, two-sided Wilcoxon Rank Sum test). Values were normalized to GAPDH.

To confirm that activation of the PI3K signaling cascade is occurring in response to expression of the induced constitutively active PI3K in these pancreatic adenocarcinomas, tumor tissue from *Pc*^*1*^ *Pik3ca*^p110*^ mice was prepared for histological sectioning and flash frozen for protein analysis. Increased phosphorylation of AKT and RPS6 was identified within pancreatic cancers by Immunohistochemistry (IHC) compared with *Pc*^*0*^ mice (Figure 2a). Immunoblotting confirmed an increase in phospho AKT and phospho RPS6 (Figure 2b). A 127% increase in phospho AKT, 144% increase in phospho RPS6 and 67% decrease in phospho 4EBP1 (Figure 2c) were measured. In association with this, increased proliferation was noted as measured by Ki67 staining (Figure 2a).

### The constitutively active PI3K in Pc^1^ Pik3ca^p110*^ induces pancreatic intraepithelial neoplasias (PanINs)

**Figure 3:**
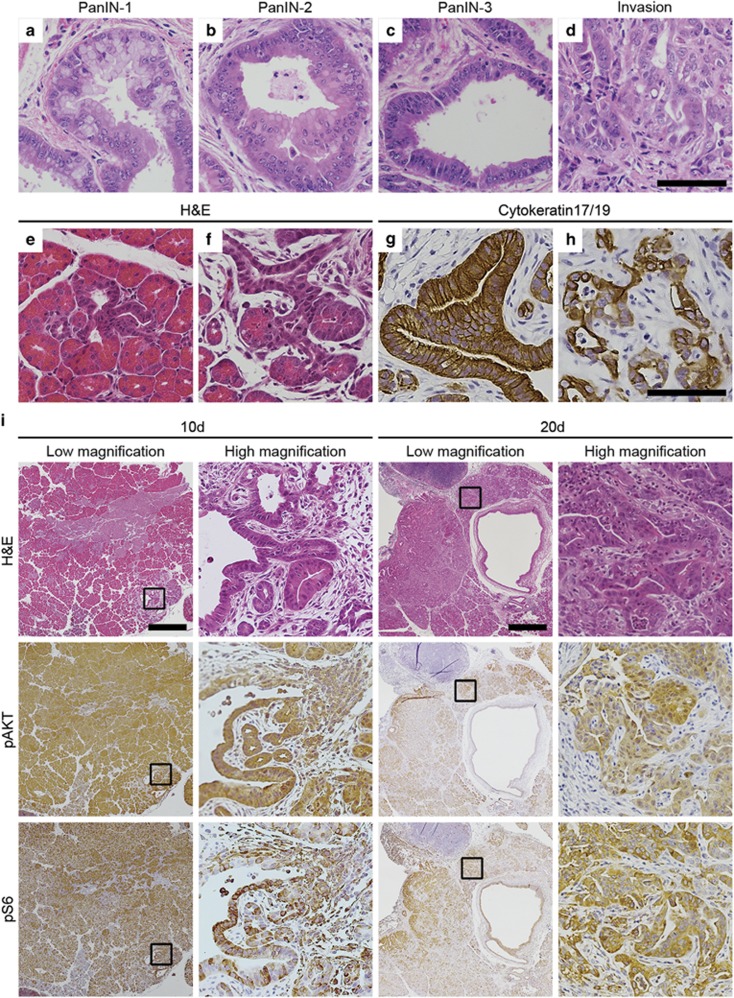
*Pc*^*1*^ *Pik3ca*^p110*^ mice develop metaplasia, PanINs and invasive cancers at very early ages. PanIN-1 (**a**), PanIN-2 (**b**), PanIN-3 (**c**) and invasive cancers (**d**) were identified commonly in *Pc*^*1*^ *Pik3ca*^p110*^ mice. Size bar for **a**–**d**, 50 μm. (**e** and **f**) At only 10 days of age cells undergoing acinar-to-ductal metaplasia were observed. (**g** and **h**) The transition to a ductal phenotype was confirmed as these cells stained positive for cytokeratin 17/19. Size bar for **e**–**h**, 50 μm, (**i**) A time course was performed starting at 10 days of age. At this time point metasplastic lesions were seen throughout the pancreas. These lesions had strong activation of the PI3K cascade as determined by increased phosphorylation of AKT and RPS6. At just 20 days of age invasive ductal adenocarcinoma was identified. These invasive lesions also had increased phsophorylation of AKT and RPS6. Size bars, 500 μm. Each high-magnification images is a × 10 enlargement of the outlined area in the low-magnification image to its immediate left.

PanINs are precursor pancreatic lesions with varying degrees of risk of progressing to invasive cancer. PanIN lesions were identified in these mice secondary to activation of PI3K signaling. PanIN-1 lesions are composed of flat or papillary columnar cells with small, round to oval, basally located nuclei (Figure 3a). PanIN-2 lesions also have flat or papillary mucinous epithelium with mildly hyperchromatic, enlarged and crowded nuclei that exhibit a loss of nuclear polarity and pseudostratification (Figure 3b). In contrast, PanIN-3 lesions frequently exhibit papillary or micropapillary architecture with back to back gland formation, prominent nucleoli, apical mitoses and a small cluster of atypical epithelium budding into the lumen of the duct (Figure 3c). In addition, the presence of invasive cancer was observed within *Pc*^*1*^ *Pik3ca*^p110*^ pancreatic tumors (Figure 3d).

### Acinar-to-ductal metaplasia occurs in Pc^1^ Pik3ca^p110*^ mice as early as 10 days of age

Acinar-to-ductal metaplasia, also known as the acinar-to-ductal transition, has been described as the process by which acinar cells of the pancreas are transformed into ductal epithelial cells.^25^ This is often seen in human pancreatic cancers.^26^ To determine whether the acinar-to-ductal metaplasia is occurring in *Pc*^*1*^ *Pik3ca*^p110*^ mice and to determine the time course over which these cancers are developing, necropsies were performed at 10 and 20 days of age. The pancreatic tissue from these mice was at least twice as abundant as compared with the pancreatic parenchyma of control mice (Supplementary Figure S2). This expansion in size was associated with a proportional increase in acinar cells staining for amylase, beta islet cells staining for glucagon and alpha islet cells staining for c-peptide (Supplementary Figure S3). Focal areas demonstrating acinar-to-ductal metaplasia were identified in all *Pc*^*1*^ *Pik3ca*^p110*^ mice (Figures 3e and i). Histological sections were stained for keratin 17/19 confirming that pancreatic cells having undergone this morphological change were now expressing markers of ductal epithelial cells (Figures 3g and h). Even at 10 days of age, the areas of metaplasia were often surrounded by a stromal reaction resembling that seen in chronic pancreatitis with abundant fibrous stroma with an infiltrate composed of lymphocytes and plasma cells (Figure 3i). These metaplastic cells demonstrate extensive staining for phospho AKT and phospho RPS6 (Figure 3i).

### Foci of invasive pancreatic adenocarcinoma are detectable in Pc^1^ Pik3ca^p110*^ mice at 20 days of age

At the 20 day time point, there was an increase in the degree of metaplasia and the surrounding stromal reaction. In addition, PanINs and invasive adenocarcinoma were identified (Figure 3i). Clusters of markedly atypical ductal cells show invasion into the stroma with an associated desmoplastic response. The adenocarcinoma cells demonstrate increased phosphorylated AKT and RPS6 as expected given the presence of the constitutively active PI3K in these cells (Figure 3i).

### The Pik3ca H1047R hotspot mutation activates the PI3K pathway and initiates the development of premalignant lesions and invasive pancreatic ductal adenocarcinoma

**Figure 4:**
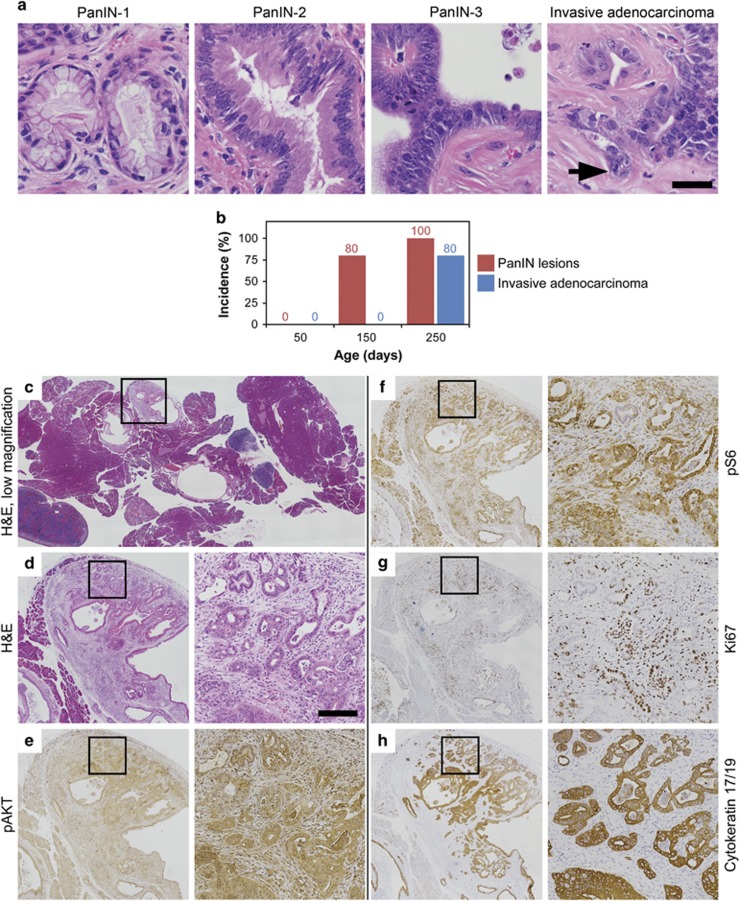
*Pc*^*1*^ *Pik3ca*^*H1047R*^ mice carry a conditional transgene encoding for the *Pik3ca* H1047R hotspot mutation. This mutation results in a constitutively active PI3K similar to that seen in the *Pc*^*1*^ *Pik3ca*^p110*^. (**a**) PanIN lesions were seen in this model in addition to invasive pancreatic ductal adenocarcinoma (arrow). Size bar, 20 μm. (**b**) This was seen with a greater latency than that observed in the *Pc*^*1*^ *Pik3ca*^p110*^ model with PanIN lesions identified at 150 days of age and most mice revealing invasive cancer by 250 days of age. (**c**) Abundant fibrous stroma was also seen. Activation of the PI3K signaling cascade was observed in these tumors, including phosphorylation of AKT (**e**) and RPS6 (**f**). Increased cellular proliferation was noted on Ki67 staining (**g**). The transition to ductal cells was confirmed with cytokeratin 17/19 staining (**h**). (**d**–**h**) Each medium-resolution photo is a × 4 enlargement of the outlined area in panel **c** (or equivalent area from the same sample); each high-resolution photo is a × 5 enlargement of the outlined area to its immediate left Size bar for high-resolution panels, 100 μm.

In human solid malignancies, mutations in the *PIK3CA* gene occur commonly in three hot spot locations, including E542K, E545K and H1047R.^27^ The H1047R mutation is the most common of the *PIK3CA* hotspot mutations across all solid tumors. To determine whether the *PIK3CA* H1047R hotspot mutation initiates tumorigenesis in pancreatic cancers similar to that observed in *Pc*^*1*^ *Pik3ca*^p110*^ mice, a total of 36 *Pc*^*1*^ *Pik3ca*^*H1047R*^ mice were generated and necropsy was performed at 50, 150 and 250 day time points. No pre-neoplastic or neoplastic pancreatic lesions were visualized on the hematoxylin and eosin-stained sections from mice at the 50 day time point. At the later time points PanIN-1, PanIN-2 and PanIN-3 lesions were observed (Figure 4a). At the 150 day time point, 80% (4/5) of the mice were observed to have PanIN lesions (Figure 4b and Supplementary Figure S4). No foci of invasion were identified at this time point. All (5/5) of the *Pc*^*1*^ *Pik3ca*^*H1047R*^ mice at the 250 day time point possessed PanINs. In addition, invasive adenocarcinoma was identified in 80% of these mice (Figures 4a and b). None of these mice became moribund out to 250 days of age. Similar to the pancreatic cancers seen in the *Pc*^*1*^ *Pik3ca*^p110*^ mice, a high degree of stromal infiltration was observed in these cancers. These lesions were surrounded by an abundant amount of normal-appearing pancreas (Figure 4c). This is in contrast to the *Pc*^*1*^ *Pik3ca*^p110*^ mice where the majority of the pancreas had undergone malignant transformation with little normal pancreas remaining. In the *Pc*^*1*^*Pik3ca*^*H1047R*^ tumors, areas of cystic dilation of the ductal epithelium were noted. Again, atypical epithelial clusters of ductal epithelium were noted to invade the stroma with some areas exhibiting single neoplastic cells. The invasive cancers observed in *Pc*^*1*^ *Pik3ca*^*H1047R*^ mice possess activation of the PI3K signaling cascade with downstream phosphorylation of AKT and RPS6 (Figures 4e and f). Non-neoplastic ducts can be seen entrapped within the carcinoma and are highlighted by their absence of staining for phospho RPS6 and Ki67 (Figures 4f and g). This is associated with increased proliferation as demonstrated by increased Ki67 staining compared with normal tissue (Figure 4g). In addition, transformation to ductal cells is demonstrated through staining for cytokeratin 17/19 (Figure 4h).

### Dual PI3K/mTOR Inhibition leads to a marked treatment response in PIK3CA mutant pancreatic cancers

**Figure 5:**
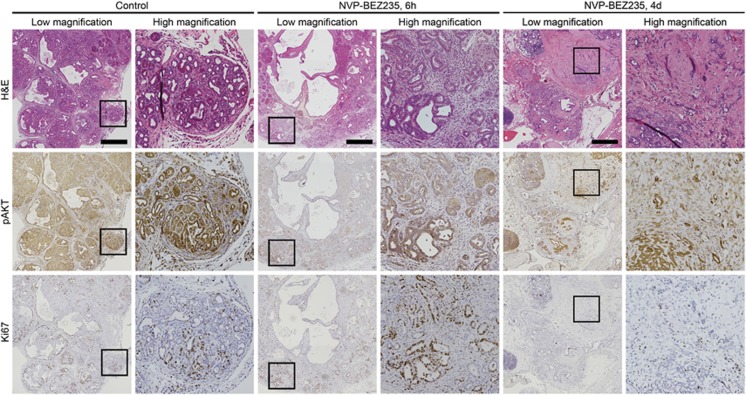
Pancreatic cancers initiated secondary to a constitutively active PI3K are profoundly sensitive to dual PI3K/mTOR inhibition. The pancreatic cancers in *Pc*^*1*^ *Pik3ca*^p110*^ mice are composed of malignant epithelial cells and dense stroma. In untreated mice, phosphorylation of AKT is observed indicating activation of the PI3K pathway (left columns). In addition, these tumors are highly proliferative with an increase in nuclear Ki67 staining (left columns). A cohort of *Pc*^*1*^ *Pik3ca*^p110*^ mice were treated with NVP-BEZ235 by oral gavage. At 6 h post treatment, a decrease in phosphorylation of AKT was identified (middle columns). After 4 days of treatment necropsy was performed and sections of the remaining pancreatic tumor were stained with H&E (right columns). A marked treatment response was observed. There was a significant decrease in the epithelial component leaving the fibrous stroma composing the majority of the remaining tumor. This was also associated with a decrease in phospho AKT and an impressive reduction in Ki67 staining. Size bar, 1mm. Each high-magnification image is a × 5 enlargement of the outlined area to its immediate left.

Inhibition of the PI3K signaling cascade continues to be an active area of both preclinical and clinical research investigations. A diverse family of inhibitors have been developed targeting multiple PI3K isoforms. Dual PI3K/mTOR inhibitors have gained significant interest given their ability to target both upstream and downstream in this signaling cascade, potentially inhibiting some feedback regulation of AKT.^28^ NVP-BEZ235 is a dual PI3K/mTOR inhibitor with an IC_50_ p110α of 4 nM, p110β of 75 nM and mTOR of 6 nM.^29^ To investigate the response of *PIK3CA* mutant pancreatic cancers secondary to PI3K pathway inhibition NVP-BEZ235 was administered to *Pc*^*1*^ *Pik3ca*^p110*^ mice. These mice were treated with NVP-BEZ235 (35 mg/kg/day dissolved in 1 volume *N*-methyl-2-pyrrolidone and 9 volumes PEG300) or vehicle-only control. Litter mates were randomly assigned to each treatment group. The investigators were not blind to the treatment cohorts. In a cohort of five *Pc*^*1*^ *Pik3ca*^p110*^ mice, the pancreas was harvested 6 h after treatment with a single dose of NVP-BEZ235. Inhibition of the PI3K cascade was demonstrated with decrease in phospho AKT (Figure 5). Proliferation in the epithelial cells of these tumors remained intact at this time point as measured by Ki67 (Figure 5). An additional cohort of four mice was treated for 4 days and the pancreas prepared for IHC and protein analysis as above. Hematoxylin and eosin staining demonstrated a profound treatment response with a marked decrease in the epithelial component such that the remaining fibrous tumor bed made up the majority of the pancreatic mass (Figure 5, far right). This was associated with a decrease in phospho AKT and a decrease in Ki67 (Figure 5, far right).

### Phosphorylation of ERK1/2 is observed in Pik3ca mutant pre-neoplastic and malignant pancreatic lesions

**Figure 6:**
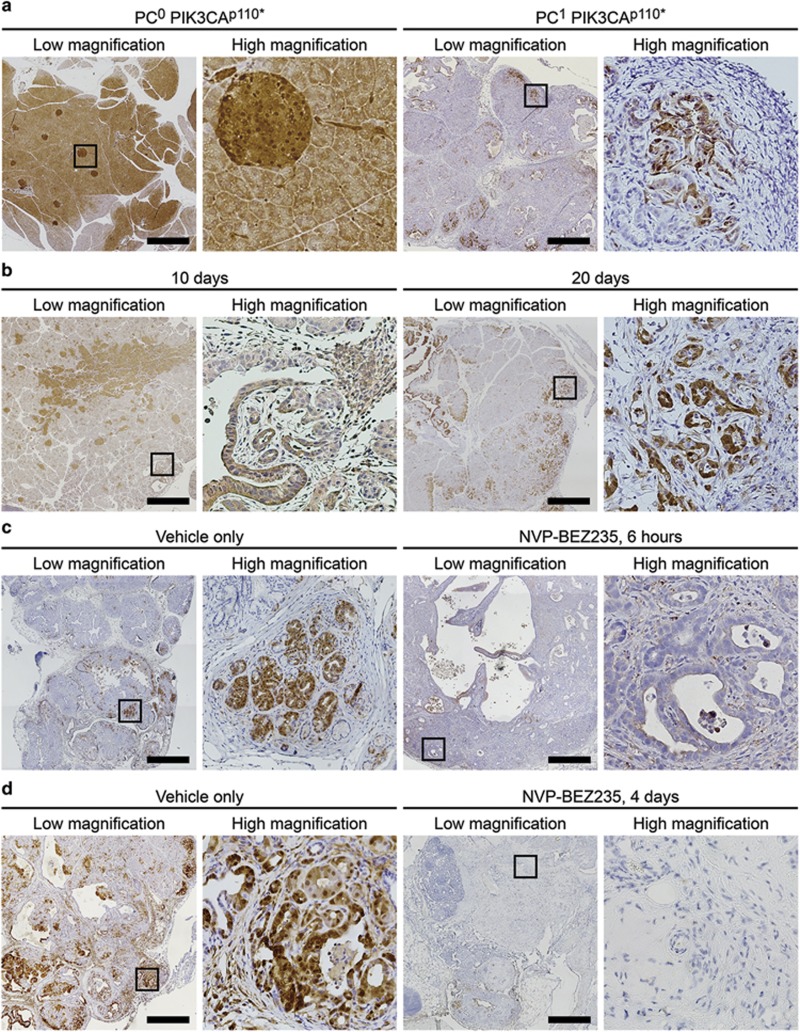
Activation of the RAS/RAF/MEK/ERK signaling cascade is a hallmark of pancreatic ductal adenocarcinoma and is present in cancers initiated by PI3K. The phosphorylation of ERK1/2 in pancreatic ductal adenocarcinoma is most commonly secondary to activating *KRAS* mutations. (**a**) The pancreatic cancers in *Pc*^*1*^ *Pik3ca*^p110*^ mice possess significant phosphorylation of ERK1/2. (**b**) Metaplastic pancreatic lesions in mice at 10 days of age and the invasive cancers in mice at only 20 days of age also demonstrate activation of ERK1/2. (**c**) The levels of phosphorylated ERK1/2 are reduced markedly 6 h after treatment of these cancers with NVP-BEZ235. (**d**) This suppression is maintained following 4 days of treatment. Size bars, 1 mm. Each high-magnification image is a × 10 enlargement of the outlined area to its immediate left.

Activation of the RAS/RAF/MEK/ERK signaling cascade is a hallmark of human pancreatic cancers.^8, 9, 10, 11, 12^ Activation of this signaling cascade in pancreatic adenocarcinoma is most commonly secondary to mutations in *KRAS* though can rarely be secondary to mutations in *BRAF.*^8^ Activation of this pathway has been implicated in resistance to agents targeting the PI3K pathway.^30, 31^ To determine whether the pancreatic cancers induced in *Pc*^*1*^ *Pik3ca*^p110*^ mice possess activation of the RAS pathway, IHC was performed to examine the phosphorylation of ERK1/2 in control mice and moribund *Pc*^*1*^ *Pik3ca*^p110*^ mice. Increased phosphorylation of ERK1/2 was demonstrated in premalignant lesions and invasive adenocarcinoma compared with control tissues and also compared with areas without invasive cancer in the *Pc*^*1*^ *Pik3ca*^p110*^ mice (Figure 6a). The foci of invasive pancreatic adenocarcinoma in *Pc*^*1*^ *Pik3ca*^p110*^ mice when moribund demonstrated increased phospho ERK1/2 staining (Figure 6a right). To examine when the activation of ERK1/2 occurs in these pancreatic cancers, tissues from the mice used for the time course, as described above, were stained for phosphorylation of ERK1/2. The metaplastic pancreatic tissue in the 10-day-old *Pc*^*1*^ *Pik3ca*^p110*^ mice demonstrated increased phospho ERK1/2 staining compared with adjacent normal tissue (Figure 6b, left). Invasive cancers seen in the 20-day-old mice also demonstrated activation of ERK1/2 (Figure 6b right).

### Phosphorylation of ERK1/2 downstream of a constitutively active PI3K is diminished in response to dual PI3K/mTOR inhibition

The ERK1/2 phosphorylation observed in precursor and neoplastic lesions might alter the response of *Pik3ca* mutant pancreatic cancers to PI3K inhibitors. However, marked responses were observed in *Pik3ca* pancreatic cancers despite this baseline ERK1/2 activation. The phosphorylation of ERK1/2 downstream of PI3K mutations has been observed in breast cancer cells with *PIK3CA* mutations.^32^ Inhibition of PI3K in these cells was actually shown to inhibit phosphorylation of ERK1/2. To ascertain if the phosphorylation of ERK1/2 persisted in *Pik3ca* mutant pancreatic cancers following treatment with a dual PI3K/mTOR inhibitor, *Pc*^*1*^ *Pik3ca*^p110*^ mice were treated with NVP-BEZ235, as above. IHC for phospho ERK1/2 of invasive pancreatic adenocarcinoma from *Pc*^*1*^ *Pik3ca*^p110*^ mice treated with vehicle alone or NVP-BEZ235 for 6 h or 4 days was performed (Figures 6c and d, respectively). A decrease in phospho ERK1/2 was seen after 6 h following a single dose of NVP-BEZ235 and was maintained following 4 days of treatment.

## Discussion

Recent successes in the use of targeted therapies have occurred when utilizing agents in cancers driven by certain oncogenes.^5, 6, 7^ Each histologic type of cancer is now understood as a collection of multiple subtypes with each individualized by its mutation profile. There remains a critical need to identify how to utilize the mutation profile to predict the benefit of these targeted therapies. Pancreatic cancer remains a deadly disease and treatment options for metastatic disease are desperately needed. Unfortunately, the use of targeted therapies for the treatment of pancreatic cancer has been limited secondary to the abundance of *KRAS* mutations. Currently, there is not an effective means to specifically target *KRAS* mutant cancers. The identification of a subtype of pancreatic cancer initiated by *PIK3CA* mutations is an exciting advance as there is great potential for patients with cancers carrying these mutations to benefit from targeted therapies. The capability to target PI3K continues to be an active area of research with clinical trials now targeting cancers with *PIK3CA* mutations.^33^ However, the patient population most likely to benefit from these therapies remains to be identified.

Investigations into the presence of *PIK3CA* mutations in pancreatic lesions have included studies examining intraductal papillary mucinous neoplasms (IPMNs), intraductal tubulopapillary neoplasm and invasive cancers. Schonleben *et al.*^34^ reported somatic mutations in *PIK3CA* in 4 out of 36 IPMNs of the pancreas. Lubezky *et al.*^35^ performed molecular profiling of 22 oncogenes in IPMN which revealed 1 *PIK3CA* mutation in 14 low grade IPMN and 1 mutation in 7 invasive IPMN. In another study, somatic mutations in exons 10 and 21 of *PIK3CA* were found in 3 of 11 intraductal tubulopapillary neoplasms. In one further study, patients with advanced cancers who were referred to a phase I program for targeted therapy, were analyzed for *PIK3CA* status.^36^ Of 217 patients, 25 were found to have *PIK3CA* mutations, of which 1 had pancreatic adenocarcinoma. Overall, *PIK3CA* mutations appear to be present in 3–5% of patients with pancreatic cancer.^20, 21, 22, 23^ Though this is a small percentage, patients with this subtype of pancreatic cancer may be exquisitely sensitive to treatments targeting the PI3K pathway. This would be a major advance owing to the grim prognosis for patients with pancreatic cancer. One patient with *PIK3CA* mutant pancreatic cancer who was treated with an inhibitor of the PI3K pathway has been described.^37^ This patient's cancer had progressed through multiple standard lines of chemotherapy, but even in the treatment-refractory setting, their cancer responded significantly to targeting the PI3K pathway. Interestingly this patient's cancer also possessed a concomitant mutation in *KRAS*. This indicates that *KRAS* mutations may not necessarily lead to resistance when targeting *PIK3CA* mutant cancers, though this needs further investigation.

Here we demonstrate that acinar-to-ductal metaplasia, PanINs and invasive pancreatic ductal adenocarcinomas can arise secondary to activating mutations in *Pik3ca*. *Pc*^*1*^ *Pik3ca*^p110*^ mice develop metaplasia surrounded by an inflammatory reaction within the pancreas by 10 days of age; these lesions progress to PanINs and even invasive ductal adenocarcinomas by 20 days of age. These large aggressive lesions result in these mice becoming moribund at a median age of only 52 days. In addition, *Pc*^*1*^ *Pik3ca*^*H1047R*^ mice, which contain the most common alteration in the *PIK3CA* gene in human cancers, were also shown to develop metaplasia, PanINs, and invasive cancer. The development of these pancreatic cancers in these models is associated with activation of AKT and RPS6. Immunoblotting for 4EBP1 demonstrated a decrease in phosphorylation. This is likely at least in part due to the high levels of phosphorylated 4EBP1 at baseline that is diminished in our protein samples due to the high degree of fibrosis and the desmoplastic reaction seen in the pancreas of these mice when moribund. A greater latency in the development of pancreatic cancers was noted in the *Pc*^*1*^ *Pik3ca*^*H1047R*^ mice likely secondary to decreased potency in the activation of the PI3K signaling cascade when comparing the *Pik3ca*^*H1047R*^ and *Pik3ca*^p110*^ alleles.

Interestingly, the *Pik3ca* mutant pancreatic cancers that develop in these models are morphologically indistinguishable from *Kras* mutant models and highly reminiscent of human cancers. This corroborates with prior investigations demonstrating the importance of PI3K in the oncogenic potential of *Kras* mutations in pancreatic cancers.^38^ These cancers also possess activation of ERK1/2 signaling similar to *Kras* models and human cancers. The phosphorylation of ERK1/2, in the models presented here, is likely downstream of PI3K signaling, as it occurs early in tumorigenesis and is diminished with PI3K/mTOR inhibition. Interestingly in *PIK3CA* mutant breast cancer cells, activation of ERK has been demonstrated and appears to be largely independent of RAS.^39^ The activation of ERK1/2 in this setting appears to instead be mediated by the RAC1/CRAF/MEK/ERK pathway. This pathway can be regulated by phosphatidylinositol 3,4,5-trisphosphate-dependent Rac exchanger 1 and the expression level of phosphatidylinositol 3,4,5-trisphosphate-dependent Rac exchanger 1 has been correlated with PI3K inhibitor sensitivity.^39^ In addition, p110α activation of RAC1 has been shown to be necessary for KRAS-mediated pancreatic tumorigenesis and p110α and RAC1 are increased in human PanINs and invasive ductal cancers.^40^

The marked responses observed in *Pc*^*1*^ *Pik3ca*^p110*^ pancreatic cancers indicate great promise for the use of agents targeting the PI3K cascade, such as dual PI3K/mTOR inhibitors. Future studies will need to further characterize the patient population with *PIK3CA* mutant pancreatic cancer, including assessment for concomitant mutations that may alter sensitivity to PI3K inhibition.

## Materials and methods

### Mouse husbandry

All animal studies were conducted under protocols approved by the Institutional Animal Care and Use Committee at the University of Wisconsin (Madison, WI, USA) following the guidelines of the American Association for the Assessment and Accreditation of Laboratory Animal Care. The Cre recombinase (*Pc*) employed in this study came from strain C57BL/6.FVB-Tg(Pdx1-cre)6Tuv/J (The Jackson Laboratory, Bar Harbor, ME, USA, stock number 014647; herein *Pc*^*1*^ denotes a carrier and *Pc*^*0*^ a non-carrier); the strain was maintained by backcrossing to C57BL/6 J mice (The Jackson Laboratory; stock number 000664). The R26Stop^FL^P110* conditional allele of *Pik3ca* (*Pik3ca*^p110*^) came from strain C57BL/6-Gt(ROSA)26Sor^tm7(Pik3ca*,EGFP)Rsky^/J (Jackson Laboratory; stock number 012343), which was maintained by crossing siblings for fewer than five generations. *Pc*^*1*^ *Pik3ca*^p110*^and *Pc*^*0*^ *Pik3ca*^p110*^ littermates were generated by crossing *Pc*^*1*^ males with *Pik3ca*^p110*^ females. The H1047R mutation of *Pik3ca* (*Pik3ca*^*H1047R+*^) came from strain FVB.129S6-Gt(ROSA)26Sor^tm1(Pik3ca* H1047R)Egan^/J (FVB.Pik3ca^H1047R^; The Jackson Laboratory; stock number 016977). F1.*Pc*^*1*^*Pik3ca*^*H1047R*^ and F1.*Pc*^*0*^*Pik3ca*^*H1047R*^ littermates were generated by crossing B6.*Pc*^*1*^ males with FVB.*Pik3ca*^*H1047R*^ females. The *mT/mG* mutation utilized to confirm the location of Cre expression came from B6.129(Cg)-Gt(ROSA)26Sor^tm4(ACTB-tdTomato,-EGFP)Luo^/J mice (B6.mT/mG; The Jackson Laboratory, stock number 007576). Genotyping was performed as previously described.^15, 24, 41, 42^

### Histology, immunohistochemistry and immunofluorescence

The pancreas was excised and fixed in 10% buffered formalin for 24–48 h. Tissues were then stored in 70% ethanol. Pancreatic tissue was embedded in paraffin, and cut into 5-μm sections. Every tenth section was stained with hematoxylin and eosin. IHC was carried out using the Histomouse Max Broad Spectrum (DAB) Kit as instructed by the manufacturer (BioCare Medical, Concord, CA, USA) except for the following modification: antigen unmasking was carried out by boiling the samples for 35 min in citrate buffer (pH 6.0). The primary antibodies included: anti-pancreatic amylase (ab21156, 1:400, Abcam, Cambridge, MA, USA), C-peptide antibody (4593, 1:100, Cell Signaling Technology, Danvers, MA, USA), glucagon antibody (2760, 1:100, Cell Signaling Technology), Ki67 (12202, 1:400, Cell Signaling Technology), phospho AKT(Ser473, 4060, 1:400, Cell Signaling Technology), phospho ERK1/2 (Thr202/Tyr204, 4370, 1:400, Cell Signaling Technology) and phospho ribosomal protein S6 (RPS6) (Ser235/236, 4858, 1:50, Cell Signaling Technology). For immunofluorescence, samples were incubated for 1 h in fluorescent secondary antibody (AlexaFlour 488 goat anti-rabbit IgG, A110088, 1:1000, Invitrogen, Grand Island, NY, USA) at room temperature and mounted with 4',6-diamidino-2-phenylindole antifade Gold mounting media (P36931, Invitrogen). Ki67 proliferation index (Ki67) was measured as the percent of Ki67-stained nuclei using the Image J Immunoratio plugin. Immunofluorescent staining of green fluorescent protein and tdTomato was performed using antigen presentation in 0.05% NaBH_4_ for 10 min and blocking with 5% non-fat dry milk for 1 h. The samples were incubated in the following primary antibodies overnight: anti-enhanced green fluorescent protein antibody (632569, 1:1000, Clontech Laboratories Inc., Mountain View, CA, USA) and anti-RFP antibody (600-401-379, 1:500, Rockland Immunochemicals Inc., Limerick, PA, USA) and then incubated with fluorescent secondary antibodies overnight (AlexaFlour 488 goat anti-mouse IgG2a, A21131, 1:1000 and AlexaFlour 568 goat anti-rabbit IgG, A11011, 1:1000, Invitrogen).

### Immunoblotting

Tissue samples were collected and flash frozen. After 24 h, protein was extracted and immunoblotting performed as previously described.^43^ The membranes were blocked with 5% non-fat dry milk for 1 h and then probed with primary antibodies against phospho RPS6 (Ser235/236, 4858, Cell Signaling Technology), phospho AKT (Ser473, 4060, Cell Signaling Technology), phospho 4EBP1 (Thr37/46, 2855, Cell Signaling Technology), total RPS6 (2217, Cell Signaling Technology), total AKT (4691, Cell Signaling Technology) or total 4EBP1(9644, Cell Signaling Technology) in bovine serum albumin at 1:1000. Anti-GAPDH antibody (8884, Cell Signaling Technology) was used as a loading control at a ratio of 1:5000.

### Dual hybrid ^18^F-FDG μPET/CT imaging

To interrogate the structural heterogeneity of pancreatic tumors in *Pc*^*1*^ *Pik3ca*^p110*^ mice, ^18^F-FDG μPET/CT imaging was utilized. All mice were fasted ~8 h and dehydrated ~2 h before ^18^F-FDG administration. Mice were intravenously injected with ^18^F-FDG and immediately anesthetized in an induction chamber using 2% isoflurane for 1 h under a heat lamp. Fasting, dehydration, isoflurane gas anesthesia and heat application are important for optimizing tumor uptake of ^18^F-FDG and increasing contrast by minimizing uptake in tissues such as brown fat, myocardium, liver, kidneys, bladder, harderian glands, skeletal muscle and the brain.^44^ All mice were imaged 1 h post ^18^F-FDG injection (~200 μCi) using the Inveon μPET/CT scanner (Siemens, Knoxville, TN, USA). Mice were scanned with a co-registered CT-based attenuation correction acquisition protocol and a subsequent 50 million count per mouse PET emission acquisition protocol. PET data were histogrammed and reconstructed using three-dimensional ordered-subset expectation maximization and maximum *a posteriori* (OSEM3DMAP), yielding superior quantitative accuracy of ^18^F due to noise reduction and increased recovery coefficient when compared with 2DFBP or OSEM2D.^45^ Analysis and images were generated using Inveon Research Workplace (Siemens) and Amira (FEI, Hillsboro, OR, USA).
