# Supplementary figures and images for: PIK3CA mutations can initiate pancreatic tumorigenesis and are targetable with PI3K inhibitors
Source: Oncogenesis. 2015 Oct 5;4(10):e169–. doi: 10.1038/oncsis.2015.28 (PMC4632089; doi:10.1038/oncsis.2015.28)

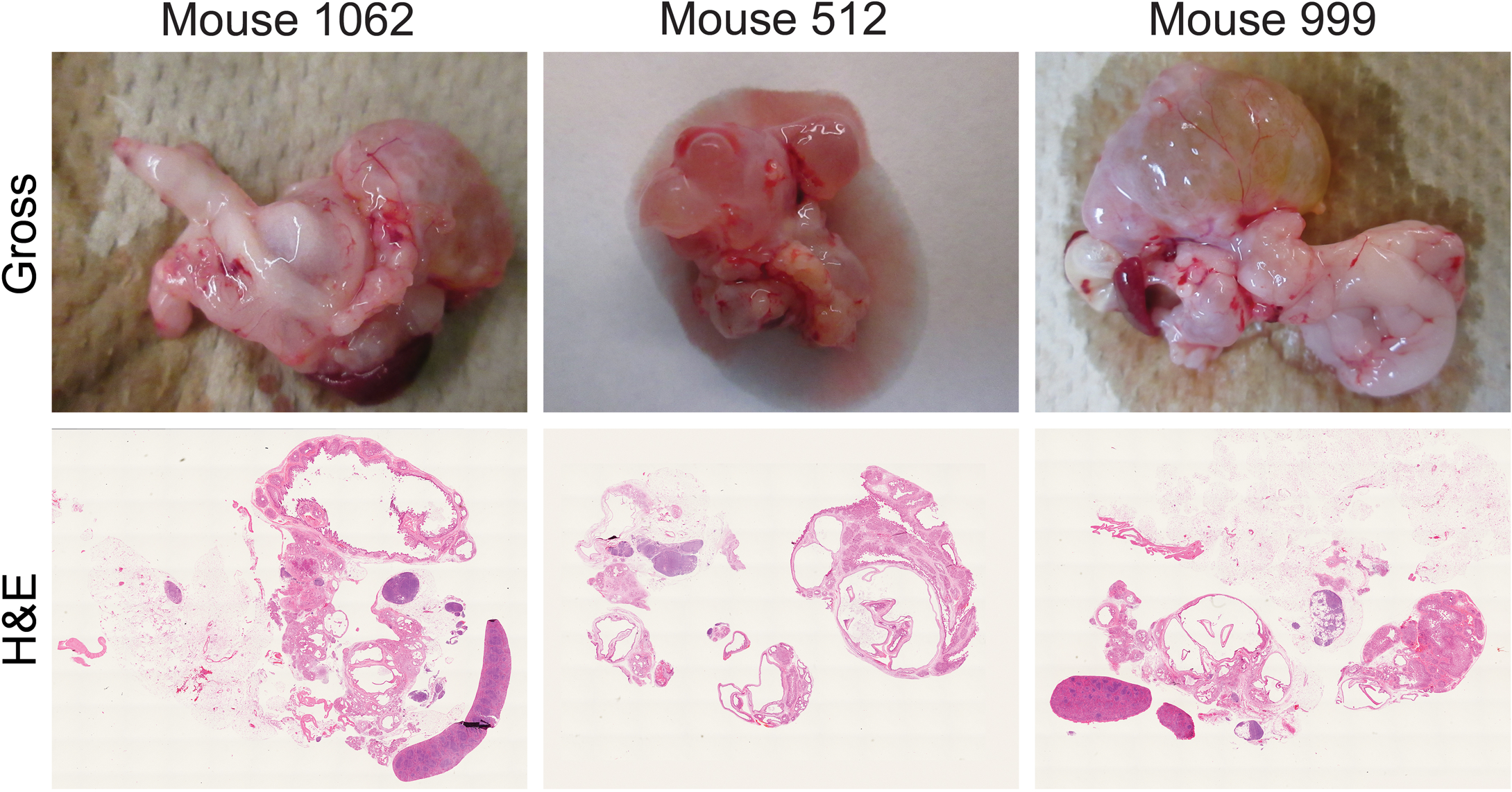

Supplement: Supplementary Figure 1 [file oncsis201528x1.tif]

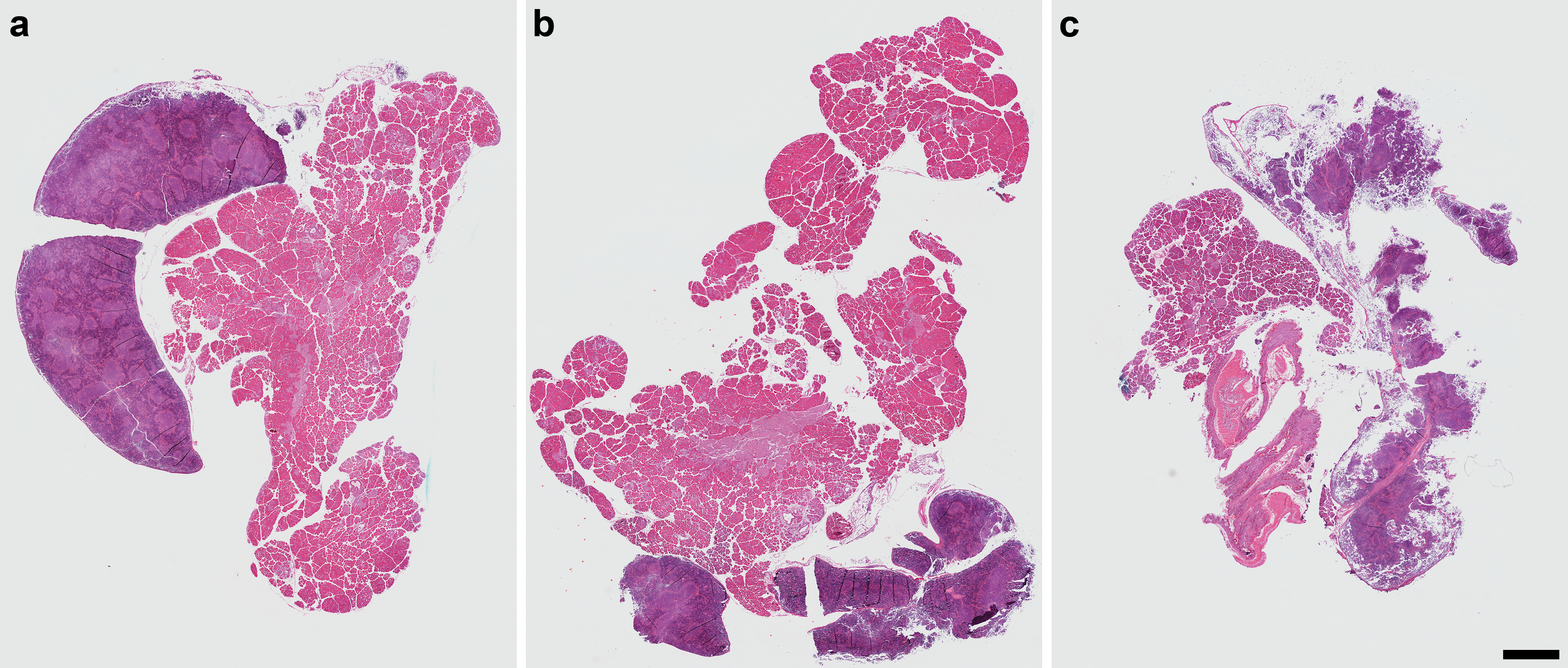

Supplement: Supplementary Figure 2 [file oncsis201528x2.tif]

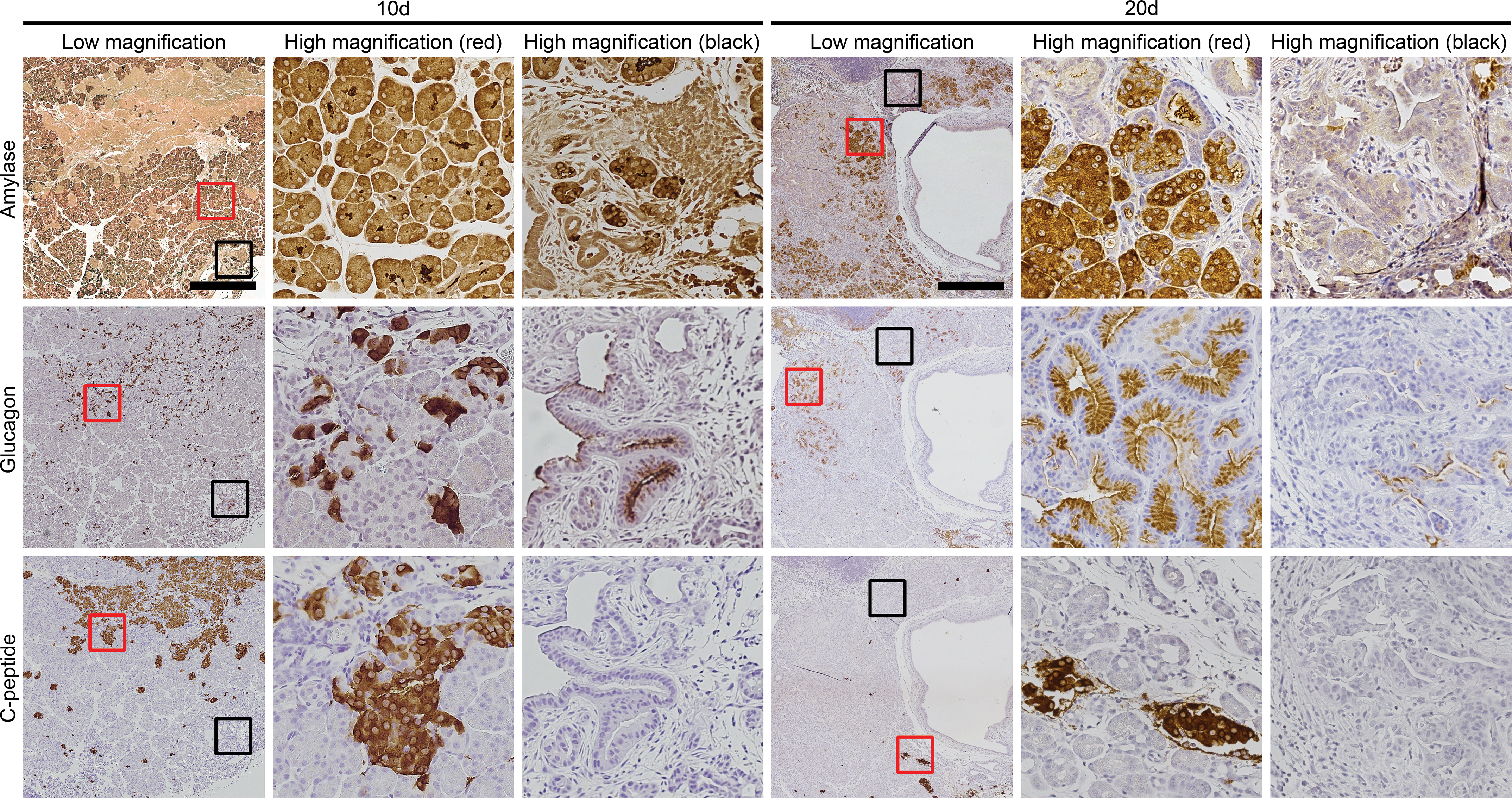

Supplement: Supplementary Figure 3 [file oncsis201528x3.tif]

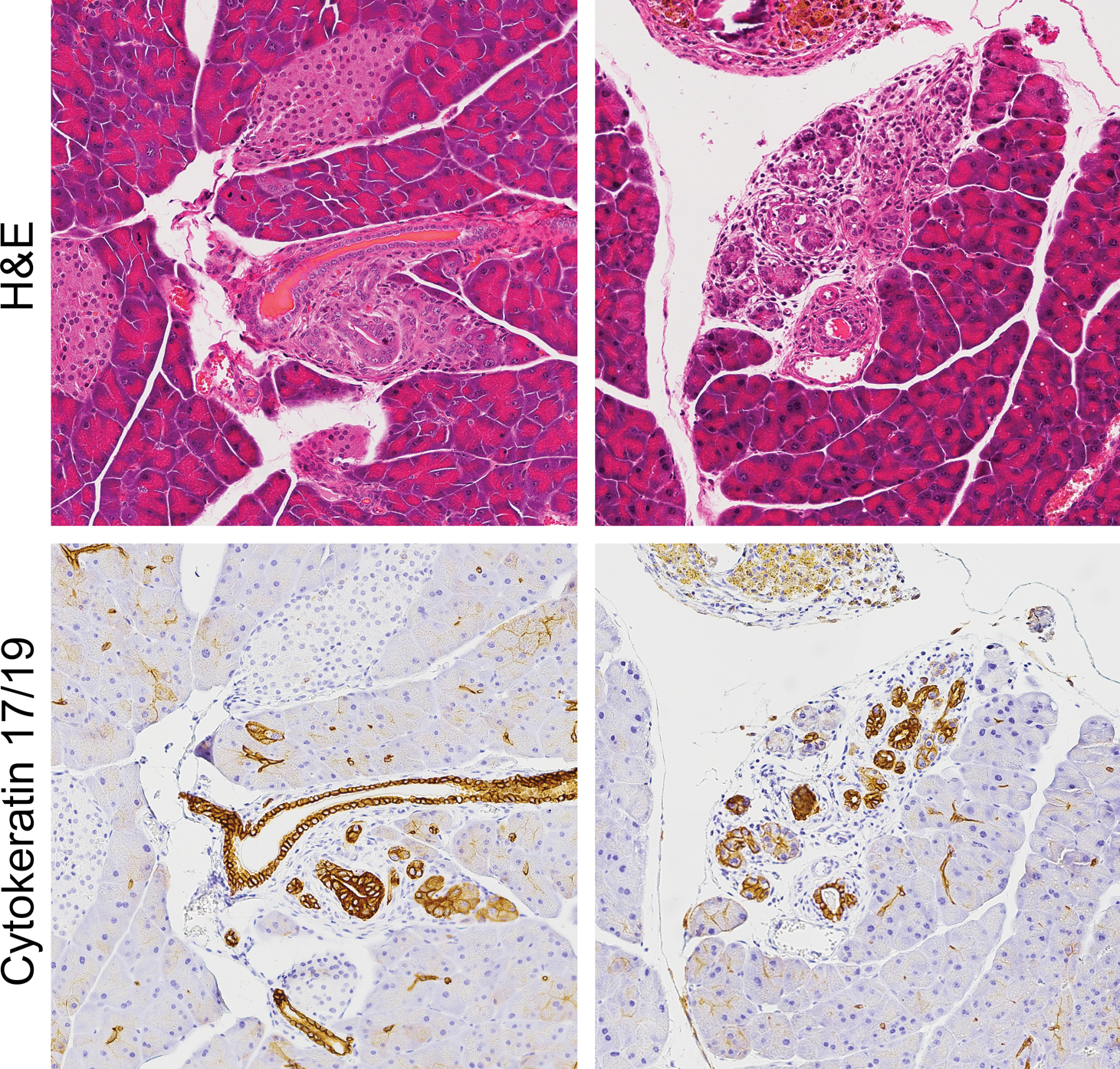

Supplement: Supplementary Figure 4 [file oncsis201528x4.tif]
