# Supplementary material for: PIK3CA mutations can initiate pancreatic tumorigenesis and are targetable with PI3K inhibitors
Source: Oncogenesis. 2015 Oct 5;4(10):e169–. doi: 10.1038/oncsis.2015.28 (PMC4632089; doi:10.1038/oncsis.2015.28)
Supplement: Supplementary Figure Legends [file oncsis201528x5.docx]

Supplementary Figure S1. Pancreatic tumors with large cystic fluid collections are common in *Pc^1^ Pik3ca^p110*^* mice. Necropsy was performed when moribund. Large cystic structures and dense fibrous components were observed in the pancreatic tumors in these mice (top row). Histological sectioning confirmed cystic dilation of the ducts surrounding areas of invasive pancreatic adenocarcinoma (bottom row).

Supplementary Figure S2. Expansion of the pancreatic parenchyma was observed in *Pc^1^ Pik3ca^p110*^* mice. Necropsy of *Pc^1^ Pik3ca^p110*^* mice was performed at 110 days of age. A dramatic difference in the size of the pancreas was noted grossly. This was confirmed upon histological sectioning. Each pancreas from the *Pc^1^ Pik3ca^p110*^* mice (**a** and **b**) was at least twice as large as those obtained from *Pc^0^ Pik3ca ^p110*^* littermates (**c**). Size bar = 1 mm.

Supplementary Figure S3. Expansion of acinar and islet cells was observed in *Pc^1^ Pik3ca^p110*^* mice at 10 and 20 days of age. Increased staining for amylase, glucagon and c-peptide was observed in the normal pancreatic tissue from *Pc^1^ Pik3ca^p110*^*mice (low magnification and high magnification red). The metaplastic lesions in the 10 day old mice and the invasive cancer in the 20 day old mice did not stain for amylase, glucagon or c-peptide (high magnification black). Size bars for low magnification images, 500 µm. Outlined areas in each low magnification image are shown enlarged 8x in the two images to its right.

Supplementary Figure S4. *Pc^1^ Pik3ca^H1047R^* mice develop metaplastic and PanIN lesions by 150 days of age. H&E staining demonstrates metaplasia and PanIN lesions in *Pc^1^ Pik3ca^H1047R^*. These lesions stain for cytokeratin 17/19 indicating a transition to a ductal phenotype.
